# Estimating Canadian sodium intakes and the health impact of meeting national and WHO recommended sodium intake levels: A macrosimulation modelling study

**DOI:** 10.1371/journal.pone.0284733

**Published:** 2023-05-10

**Authors:** Nadia Flexner, Anthea K. Christoforou, Jodi T. Bernstein, Alena P. Ng, Yahan Yang, Eduardo A. Fernandes Nilson, Marie-Ève Labonté, Mary R. L’Abbe

**Affiliations:** 1 Department of Nutritional Sciences, University of Toronto, Toronto, Ontario, Canada; 2 Center for Epidemiological Research on Health and Nutrition, University of São Paulo, São Paulo, State of São Paulo, Brazil; 3 Centre Nutrition, Santé et Société (NUTRISS), Institute of Nutrition and Functional Foods (INAF), Laval University, Quebec City, Quebec, Canada; PAHO/WHO, UNITED STATES

## Abstract

**Background:**

Cardiovascular diseases (CVDs) are the second leading cause of total deaths in Canada. High blood pressure is the main metabolic risk factor for developing CVDs. It has been well established that excess consumption of sodium adversely affects blood pressure. Canadians’ mean sodium intakes are well above recommended levels. Reducing dietary sodium intake through food reformulation has been identified as a cost-effective intervention, however, dietary sodium intake and the potential health impact of meeting recommended sodium intake levels due to food reformulation have not been determined in Canada.

**Objective:**

This study aimed to 1) obtain robust estimates of Canadians’ usual sodium intakes, 2) model sodium intakes had foods been reformulated to align with Health Canada’s sodium reduction targets, and 3) estimate the number of CVD deaths that could be averted or delayed if Canadian adults were to reduce their mean sodium intake to recommended levels under three scenarios: A) 2,300 mg/d–driven by a reduction of sodium levels in packaged foods to meet Health Canada targets (reformulation); B) 2,000 mg/d to meet the World Health Organization (WHO) recommendation; and C) 1,500 mg/d to meet the Adequate Intake recommendation.

**Methods:**

Foods in the University of Toronto’s Food Label Information Program 2017, a Canadian branded food composition database, were linked to nationally representative food intake data from the 2015 Canadian Community Health Survey–Nutrition to estimate sodium intakes (and intakes had Health Canada’s reformulation strategy been fully implemented). The Preventable Risk Integrated ModEl (PRIME) was used to estimate potential health impact.

**Results:**

Overall, mean sodium intake was 2758 mg/day, varying by age and sex group. Based on ’reformulation’ scenario A, mean sodium intakes were reduced by 459 mg/day, to 2299 mg/day. Reducing Canadians’ sodium intake to recommended levels under scenarios A, B and C could have averted or delayed 2,176 (95% UI 869–3,687), 3,252 (95% UI 1,380–5,321), and 5,296 (95% UI 2,190–8,311) deaths due to CVDs, respectively, mainly from ischaemic heart disease, stroke, and hypertensive disease. This represents 3.7%, 5.6%, and 9.1%, respectively, of the total number of CVDs deaths observed in Canada in 2019.

**Conclusion:**

Results suggest that reducing sodium intake to recommended levels could prevent or postpone a substantial number of CVD deaths in Canada. Reduced sodium intakes could be achieved through reformulation of the Canadian food supply. However, it will require higher compliance from the food industry to achieve Health Canada’s voluntary benchmark sodium reduction targets.

## Introduction

Cardiovascular diseases (CVDs) are the leading cause of mortality worldwide and the main cause of disability–mainly with regards to ischemic heart disease (IHD) and stroke [1]. High blood pressure, a major public health issue, remains a crucial metabolic risk factor for developing CVDs [1]. Worldwide, 1.28 billion adults (30–79 years) are affected by high blood pressure, doubling the number of people affected since 1990 [2]. It has been well established that excessive consumption of sodium adversely affects blood pressure [3–9]. Worldwide, individuals consume too much sodium (between 3,600–4,800 mg/day of sodium) [10]. The World Health Organization (WHO) recommends sodium intakes of less than 2,000 mg/day to prevent non-communicable diseases (NCDs) [10]. In 2019, the National Academies of Sciences, Engineering and Medicine published the Updated Dietary Reference Intakes for Sodium and Potassium, where sodium Adequate Intakes (AI) for adults were established at 1,500 mg/day and the recommendation for Chronic Disease Reduction Intake (CDRR) was set at 2,300 mg/day [11]. Health Canada’s sodium intake recommendations for adults [12, 13] are consistent with these recommendations.

CVDs are the second leading cause of death in Canada, after cancers, and account for almost 25% of all deaths annually [14]. High blood pressure affects almost 1 in 4 Canadian adults [15]. Moreover, dietary sodium intake levels remain high, with six out of ten Canadians consuming too much sodium on a daily basis [13]. Canadians consume an average of 2,760 mg of sodium per day [13], which is well above national and international recommended levels [10–12]. Canadian children, adolescents and adult males are the population sub-groups that significantly exceed the recommended sodium intake levels [13]. These estimates, however, may be underestimated, particularly across certain age and sex groups, given known limitations in the accuracy of generic food composition databases conventionally used to examine dietary intakes [16] and recent evidence documenting significant increases in the sodium content of some key sodium contributing food categories across the Canadian food supply [17].

Canada has committed to reaching the WHO’s 30% relative reduction in mean population intake of sodium by 2025 [18], which would bring the mean sodium intakes of Canadians–from a baseline of 3,400 mg/d (CCHS 2004) [19]–to recommended CDRR levels (2,300 mg/day) [11]. Reducing dietary sodium intake is a cost-effective intervention that can save many lives by preventing and decreasing the burden of diet-related NCDs [20]. Evidence suggests that every US $1 invested in reducing dietary sodium intake at the population level will yield a return of investment of between US $13 and $19 [21, 22]. Furthermore, food reformulation has been identified by the WHO as one of the most cost-effective interventions for addressing the current burden of diet-related NCDs [20, 23], including CVDs. Food product reformulation can be defined as the lowering of levels of critical nutrients in packaged food products (e.g. sodium, sugars, saturated fat, or trans fat) while maintaining flavor, texture and shelf life [24, 25]. These initiatives have been implemented in several countries by governments or the food industry on either a voluntary or mandatory basis [24, 26–28].

Food reformulation efforts in Canada have primarily been implemented on a voluntary basis. The *Sodium Reduction Strategy for Canada* (2010) included comprehensive recommendations to reduce population sodium intakes. These recommendations focused on the following areas: sodium levels in the food supply, consumer education and awareness, research related to sodium reduction initiatives, and monitoring and evaluation [19]. In 2012, Health Canada published a set of voluntary benchmark sodium reduction targets to guide food industry efforts in achieving product reformulation by 2016 [29]. These targets were developed in conjunction with the food industry, health sector, and research experts and aimed to achieve gradual sodium reduction in processed foods while ensuring food product quality, food safety, and consumer acceptance [29]. Evaluations of this initiative has shown only modest results [30, 31]; by 2017, only 13 of 94 (14%) food categories met the 2016 sodium reduction targets [30]. Recently, in December 2020, Health Canada updated the voluntary sodium reduction targets for processed foods, adjusting some food categories and expanding the number of food categories from 94 to 117 [32]. Similarly, the United Kingdom updated voluntary sodium reduction targets for processed foods (2020–2025) for 84 processed food categories [33], and at the global level, in May 2021, the WHO launched the *“WHO global sodium benchmarks for different food categories”*, which includes target levels for 18 overall food categories and 97 food subcategories [34].

Policymakers are increasingly reliant on health and economic analysis to support the decision-making process, in most cases using simulation modelling methods as a tool for policy support and prioritization [35, 36]. Given the challenges of performing traditional epidemiological research to measure the health and economic impact of public health policies (such as cohort studies, randomized controlled trials or natural experiments), simulation modelling methods are used as a suitable and strategic tool with which to estimate the impact of a policy before actual policy implementation [36–39]. For instance, there is considerable evidence in the scientific literature on simulation modelling studies that estimated the impact of food reformulation strategies on health or nutrient intakes [38], and sodium is a critical nutrient studied most often.

Modelling approaches vary from each other in complexity, structure, health and economic perspective, epidemiological data, reporting quality, the inclusion or not of a time component (dynamic or static), outcome measures, and assumptions [38, 40]. When selecting an epidemiological simulation model, several factors are considered: the research question, resources, stakeholders needs, data needs/availability and quality, and model structure [40]. For this study we used the Preventable Risk Integrated ModEl (PRIME) [39], a comparative risk assessment model.

One earlier study examined the health impact of reducing Canadian sodium intakes from 3400 to 2300 mg/day [41]. To the best of our knowledge, the potential health impact of further changes in sodium intake resulting from meeting Health Canada’s sodium reduction targets for processed foods, and other more stringent recommended sodium intake levels remains unknown in the Canadian context. Thus, the objectives of the current study were to 1) obtain robust estimates of Canadian’s usual sodium intakes, by linking the Food Label Information Program (FLIP) 2017, a database of Canadian branded food and beverage products, to food intakes reported in the 2015 Canadian Community Health Survey (CCHS)–Nutrition, 2) model sodium intakes had foods been reformulated to align with Health Canada’s sodium reduction reformulation targets for processed foods, and 3) estimate the number of CVD deaths that could be averted or delayed if Canadian adults were to reduce their mean sodium intake to recommended levels under three scenarios: A) 2,300 mg/d, driven by a systematic reduction of sodium levels in packaged foods (reformulation); B) 2,000 mg/d, to meet the World Health Organization (WHO) recommendation; and C) 1,500 mg/d, to meet the Adequate Intake recommendation.

## Materials and methods

### Dietary data: CCHS–Nutrition 2015

Data from the CCHS-Nutrition 2015 [42] were used to estimate Canadian’s sodium intakes. CCHS-Nutrition-2015 is a nationally representative, cross-sectional food and nutrition survey that uses a 24-hour dietary recall and is conducted via computer-assisted in-person interviews by trained personnel. It collected data on 20,487 individuals, with an additional 1-day recall conducted via telephone on 35% of the sample. This analysis included all respondents (≥1 year of age) from the CCHS-Nutrition 2015 sample, excluding pregnant and breastfeeding women and respondents with null intakes, which left a total of 20,176 participants included for the overall sodium intake estimations and 13,612 adults ≥19 yrs for the specific objectives of this study.

Sodium and calorie content of each food item (the latter was used to ascertain energy misreporting status) were imported into CCHS-Nutrition from a Canadian branded prepackaged food composition database, the University of Toronto’s Food Label Information Program database (FLIP)– 2017. This nutritional content information was merged with the *Food and Ingredient Details* (FID) file used in the CCHS-Nutrition 24-hour recall by matching FLIP food products with equivalent CCHS-Nutrition food profiles, the detailed methods of which have been discussed elsewhere [16]. Linking food composition data from the FLIP dataset onto generic foods reported by participants via 24-hour recalls in the CCHS ensures nutritional composition was based on detailed and systematically collected data of grocery items to maximize the accuracy of dietary estimates.

The FLIP 2017 database includes information from the Nutrition Facts Table (NFt), ingredients list, product price, barcodes and photos of all sides of the product packaging [43, 44] for over 17,000 unique food and beverage products. Data collection took place at the largest grocery retailers and represented approximately 65–75% of the Canadian food retail market share, ensuring that most products available to Canadian consumers were captured. The linkage of large, branded food supply data with intake data also allows for scenario modelling whereby foods in the FLIP database were “reformulated” to meet target formulations, based on dietary policies, to assess the impact on diets and health. To the best of our knowledge, we are the only research group to apply such a linkage.

### Baseline scenario: Canadians’ actual sodium intake

For the baseline scenario, we estimated Canadians’ age- and sex-specific sodium intakes using linked food composition data [16] gleaned from the FLIP 2017 dataset of Canadian brand-name prepackaged foods. The methods for this linkage have been described in detail elsewhere [16]. Briefly, generic foods from the FID file in the CCHS-Nutrition were matched to equivalent packaged food products in the FLIP 2017 database to create aggregate food profiles (i.e. mean sodium value for a specific food category) based on FLIP 2017 nutrient data. Where a FLIP 2017 product was not matched to an FID food profile such as unpackaged food item (e.g. whole fruit and vegetables) or where there was no appropriate match (e.g. FLIP 2017 product that is chia pudding but no equivalent listed in the FID food file) nutrient composition information was left at that reported in the FID file.

Canadians’ sodium intakes (mean, SE) were stratified by Dietary Reference Intakes (DRI) age/sex groups and compared to their respective AI and CDRR levels (i.e., percent within each age group below the AI and above the CDRR, respectively) using the DRI cut point method [45].

### Counterfactual scenarios: Food reformulation scenario for meeting Canadian reformulation targets and the WHO and AI recommended sodium intake levels

#### Scenario A

For the ‘reformulation’ scenario, we included Canadians’ age- and sex-specific sodium intake after modelling a systematic reduction in the sodium content of processed foods in Canada to meet Health Canada’s 2016 sodium reduction targets [29]. All products in FLIP 2017 were categorized into sodium reduction food group categories based on those outlined in Health Canada’s *Guidance for the Food Industry on Reducing Sodium in Processed Foods* (Health Canada 2012) [29], independently by two study authors. Where disagreements arose a third author was inlisted to resolve discordant categorization. Foods in FLIP 2017 with reported sodium levels above sodium reduction targets for their respective categories were ‘reformulated’ or given values at the target sodium level. ‘Reformulated’ scenario values were imported into CCHS-Nutrition, as described above, to model usual sodium intakes had all foods available for sale in Canada adhered to the voluntary sodium reduction policy. ‘Reformulation’ scenario sodium intakes (means, SE) were also stratified by DRI age/sex group and the proportion of respondents within each group below their respective AI and CDRR levels estimated.

#### Scenarios B and C

The counterfactual scenarios of meeting the WHO (2,000 mg/d) and AI (1,500 mg/d) sodium recommended levels were estimated by first calculating the proportion to be reduced from the baseline scenario–Canadians’ actual mean sodium intake–and then by applying the same proportion to each DRI age/sex group. WHO and AI’s sodium intake recommendations are directed to individuals and not to the population average. However, given that some age groups in Canada are already consuming less than the sodium recommended levels, and country monitoring progress is usually presented at the population level, we decided to test counterfactual scenarios aiming to achieve recommended sodium intake at the population level.

As PRIME requires standard deviations, these were estimated for the baseline and ‘reformulation’ scenario (scenario A) using standard errors calculated through usual intake models for sodium described below. For scenarios B and C, it was assumed that the SD represented the same proportion of the mean as in the baseline scenario for each DRI age/sex group.

### Health impact modelling

For this study, we employed the cross-sectional NCD scenario model PRIME [39], to estimate the number of CVD deaths that could have been averted or delayed, due to reducing Canadians’ mean sodium intake to recommended levels under the three scenarios described above. The data requirements of the model include the following age- and sex-specific sets of data from the population under study: 1) estimates of the number of individuals in the population; 2) estimates of the annual number of NCD deaths relevant to the study; 3) population distribution of behavioral risk factors (baseline scenario) and the counterfactual distribution of behavioral risk factors being studied (counterfactual scenarios).

PRIME is a macrosimulation NCD scenario model that has been widely used in other countries [46–64], including Canada [41, 65], and applied to different scenarios of public health initiatives [46–64]. PRIME estimates the impact of population-level changes in the distribution of behavioral risk factors on population NCD mortality [39]. The model specifically answers the question *“How many deaths would have occurred in the baseline year if the distribution of risk factors had been different*? [66].*”* PRIME is an open access and user-friendly NCD scenario modelling tool available through the WHO Regional Office for Europe web portal [66] or from its authors at the University of Oxford upon request. PRIME includes twelve behavioral risk factors, including alcohol consumption, smoking, physical activity, and diet (energy, fruits & vegetables, fiber, salt, total fat, saturated fat, unsaturated fat and cholesterol), as well as twenty-four health outcomes including CVDs, cancers, diabetes, kidney disease, liver disease and chronic obstructive pulmonary disease [39]. The impact on the health outcomes due to a change in one or more of the behavioral risk factors is modelled directly or via one of three intermediate risk factors: blood pressure, serum blood cholesterol, or BMI [39].

The model parameterizes the links between the behavioral risk factors and NCD mortality using robust meta-analyses taken from epidemiological studies [39]. PRIME more specifically estimates the effect of sodium intake reduction on CVDs outcomes through the parametrization of sodium intake and changes in systolic blood pressure [4], and consequently changes in blood pressure and CVDs outcomes [67]. In other words, we estimated the number of CVD deaths that could be averted or delayed due to changes in blood pressure (intermediate risk factor) resulting from changes in population sodium intakes (baseline and counterfactual scenarios described above).

### Population demographics and CVDs mortality burden

Population demographics and the most recent available data (2019) on mortality associated with CVDs (ischaemic heart diseases, cerebrovascular disease, heart failure, aortic aneurysm, pulmonary embolism, rheumatic heart disease, and hypertensive disease)—stratified by sex and five-year age band, from 20 to over 85 years of age—were obtained from the publicly available Statistics Canada CANSIM tables [68, 69] (S1, S2 Tables in S1 File). We included the most recent CVD mortality data assuming no major changes in Canadians’ sodium intake between 2015 (last CCHS-Nutrition survey) and 2019. Mortality data were based on the WHO International Classification of Diseases 10 (ICD 10) [70].

### Statistical analyses

Usual sodium intake, stratified by DRI age/sex group, under the actual (baseline) and ‘reformulation’ scenario (scenario A), were estimated using both available 24-hour recall days from CCHS-Nutrition 2015. The National Cancer Institute (NCI) method [71] was used to estimate usual intake distributions of sodium. NCI usual intake models controlled for sequence of recalls, whether the recall was carried out for a weekday or weekend day as well as misreporting status [71, 72].

The level of misreporting was defined as a categorical variable with *under-reporters* defined as those with reported calorie intakes at or less than 70% of their estimated energy requirements (EER) [73], *over-reporters* with reported calorie intakes more than 142% of their EER, and *plausible reporters* identified as those with reported calorie intakes between 70% and 142% of their EER [74]. The Institute of Medicine’s equations for predicted energy requirements [73] were used when measured height and weight or adult self-reported height and weight was available in CCHS-Nutrition 2015. Adult self-reported heights and weights were adjusted using an established correction factor, given known biases associated with these measures in the Canadian population [75]. Where height and weight data were not available for certain respondents, estimated energy needs for reference heights and weights were applied using USDA estimates [76]. Physical activity levels for adults were classified as sedentary (i.e. no moderate or vigorous activity or <30 minutes activity per day), low-active (i.e. 30–60 minutes per day), active (i.e. 60–180 minutes per day), or very active (i.e. >180 minutes per day) [42]. For children 14 to 19 years, physical activity levels were coded as ‘sedentary’ and ‘low-active’ for those 13 years and under, based on previous reports on physical activity levels in Canadian children [77].

Balanced repeated replication with 500 replicates was used to estimate all standard errors using bootstrap weights provided by Statistics Canada. All analyses were survey-weighted using the master sample survey weights provided by Statistics Canada to allow for representative estimates and were conducted at the Statistics Canada Research Data Centre at the University of Toronto.

For the scenario modelling, all required data was inputted in PRIME. The model estimated changes in the annual number of deaths attributable to CVDs between the baseline and each counterfactual scenario. Monte Carlo analysis, built into PRIME, was performed at 10,000 iterations to estimate 95% uncertainty intervals (UIs) around the results (based on 2.5^th^ and 97.5^th^ percentiles). This allowed the epidemiological parameters to vary randomly according to the distributions reported in the literature [39]. For each scenario tested, PRIME estimated the CVD deaths that could have been averted or delayed for the overall population and disaggregated by sex and type of CVD.

## Results

### Usual sodium intakes

Mean sodium intakes, using linked mean aggregate sodium values obtained through the FLIP 2017 branded food database, were 2758 mg/day, ranging from 2236 mg/day amongst 19–30-year-old females to 3196 mg/day amongst males 71 years of age or older **(Table 1)**. On average, only 9.4% of Canadians had daily intakes of sodium at or below the AI level and 62.9% had intakes above the CDRR **(Table 1)**. These values also ranged considerably by age and sex groups with females aged 19–30 years having a higher proportion of respondents with intakes below the AI for sodium at 20.9% and the fewest (42.4%) above the CDRR **(Table 1)**.

**Table 1:** Usual sodium intake (mg/day) among Canadians, calculated using data from CCHS-Nutrition 2015 national survey linked to FLIP 2017 branded food composition data, as compared to the sodium Dietary Reference Intake values, overall (n = 20,176) and by age/sex groups (n = 13,612).

Sex	Age	n	Mean (SE) (mg/day)	25^th^ (SE) (mg/day)	50^th^ (SE) (mg/day)	75^th^ (SE) (mg/day)	AI (mg/day)	Below AI (%)	CDRR (mg/day)	Above CDRR (%)
Total	1–71+	20176	2758 (21)	1991 (19)	2627 (20)	3381 (29)	-	9.4 (0.5)	-	62.9 (0.7)
**Male**	19–30	882	2983 (64)	2204 (53)	2850 (68)	3619 (79)	1500	4.3 (0.7)	2300	71.3 (2.2)
	31–50	2077	3036 (42)	2285 (40)	2933 (46)	3660 (50)	1500	3.5 (0.5)	2300	74.9 (1.5)
	51–70	2246	3103 (44)	2288 (39)	2958 (47)	3760 (53)	1500	3.1 (0.4)	2300	74.5 (1.4)
	71+	1246	3196 (45)	2414 (42)	3095 (48)	3846 (53)	1500	2.4 (0.4)	2300	79.0 (1.4)
**Female**	19–30	897	2236 (48)	1591 (38)	2126 (51)	2756 (61)	1500	20.9 (1.6)	2300	42.4 (2.3)
	31–50	2288	2333 (30)	1684 (30)	2236 (33)	2857 (36)	1500	17.4 (1.1)	2300	47.0 (1.5)
	51–70	2420	2467 (28)	1798 (29)	2371 (29)	3000 (33)	1500	13.6 (0.9)	2300	53.1 (1.3)
	71+	1556	2525 (39)	1838 (38)	2424 (40)	3081 (45)	1500	12.6 (1.1)	2300	55.4 (1.7)

The National Cancer Institute (NCI) method was used to obtain usual intake distributions by Dietary Reference Intake (DRI) age and sex groups. All analyses controlled for sequence of recalls (i.e. respondents 1^st^ or 2^nd^ day of dietary information), day of the week and misreporting status. M = males; f = females; SE = Standard Error; AI = Adequate Intake; CDRR = Chronic Disease Risk Reduction Intake; mg/d = milligrams per day; FLIP = Food Label Information Program.

### Sodium intakes based on reformulated food supply modelling

Canadian usual sodium intakes based on a ‘reformulation’ scenario (scenario A), whereby food and beverages were ‘reformulated’ to comply with Health Canada’s 2016 voluntary sodium reduction benchmark targets, were reduced on average by 459 mg/day, to 2299 mg/day **(Table 2)**. The proportion of Canadians with sodium intakes below the AI and above the CDRR also improved to 17.7% and 45.0%, respectively–an improvement of 88% and 50.0%, respectively. Results from the ‘reformulation’ scenario of sodium intakes were lower than actual intakes across age and sex groups **(Table 2 compared to Table 1)**. Sodium intakes based on meeting WHO recommendations (2000 mg/day) and AI (1500 mg/day) are shown in **Table 3**, and were used for the health impact calculations.

**Table 2:** Canadian mean usual sodium intakes (mg/day)^1^ if all foods met Health Canada’s sodium reduction targets designed to meet population mean intake of 2,300 (mg/day) (Reformulation Scenario A), compared to sodium Dietary Reference Intake values, overall (n = 20,176)and by adult age/sex group (n = 13,612).

Sex	Age	Mean (SE) (mg/day)	Below AI (%)	Above CDRR (%)
Total	1–71+	2299 (12)	17.7 (0.2)	45.0 (0.2)
**Male**	19–30	2485 (40)	10.7 (1.2)	53.6 (2.3)
	31–50	2529 (17)	2.4 (0.4)	56.8 (1.7)
	51–70	2585 (21)	8.4 (1.3)	57.5 (1.9)
	71+	2662 (13)	6.5 (0.8)	62.7 (1.5)
**Female**	19–30	1866 (24)	34.2 (1.4)	24.8 (1.2)
	31–50	1948 (50)	29.6 (1.9)	28.4 (2.9)
	51–70	2059 (12)	24.4 (0.7)	33.7 (0.7)
	71+	2108 (29)	22.8 (1.3)	36.5 (1.5)

^1^ ‘Reformulation’ scenario values are modeled on sodium values for packaged foods in line Health Canada’s 2016 voluntary Sodium Reduction Targets, see methods for full details on how ‘reformulation’ scenarios were simulated. The National Cancer Institute (NCI) method was used to obtain usual intake distributions of sodium by Dietary Reference Intake (DRI) age and sex groups. All analyses controlled for sequence of recalls (i.e., respondents 1^st^ or 2^nd^ day of dietary information), day of the week and misreporting status. M = males; f = females SE = Standard Error; AI = Adequate Intake; CDRR = Chronic Disease Risk Reduction Intake; mg/d = milligrams

per day.

**Table 3:** Mean sodium intakes (mg/day) of Canadian adults at baseline (current) and according to sodium reduction scenarios based on: A) a systematic reduction of the sodium content in packaged foods to meet population mean sodium intake target (2,300 mg/d); B) meeting the WHO recommendation (2,000 mg/d); and C) meeting the Adequate Intake (AI) recommendation for adults (1,500 mg/d).

Sex	Age	n	Baseline current mean sodium intake (SE)	Baseline SD ^1^ current mean sodium intake (mg/d)	Scenario A ^3^ Reformulation mean sodium intake (SE) (2300 mg/d)	Scenario A SD ^2^ Reformulation sodium intake (mg/d)	Scenario B ^3^ WHO goal mean sodium intake (2000 mg/d)	Scenario B SD ^2^ WHO goal sodium intake (mg/d)	Scenario C ^3^ AI goal sodium intake (1500 mg/d)	Scenario C SD ^2^ AI goal sodium intake (mg/d)
**Total**	1–71+	20176	2758 (21)	2997	2299 (12)	1733	2000	2174	1500	1630
**Male**	19–30	882	2983 (64)	1886	2485 (40)	1200	2163	1367	1623	1026
	31–50	2077	3036 (42)	1910	2529 (17)	757	2201	1384	1652	1039
	51–70	2246	3103 (44)	2081	2585 (21)	991	2249	1508	1688	1132
	71+	1246	3196 (45)	1596	2662 (13)	455	2317	1157	1739	868
**Female**	19–30	897	2236 (48)	1438	1866 (24)	719	1621	1042	1216	782
	31–50	2288	2333 (30)	1445	1948 (50)	2373	1692	1047	1269	786
	51–70	2420	2467 (28)	1373	2059 (12)	585	1788	995	1342	747
	71+	1556	2525 (39)	1519	2108 (29)	1144	1831	1101	1374	826

^**1**^ SD for current mean sodium intake and scenario A (reformulation) was calculated using SE estimations.

^**2**^SD estimation for scenario B and C assumed to represent the same proportion (%) of the mean as in the current mean sodium intake (CCHS-Nutrition 2015).

^**3**^Approximately a 17%, 28% and 46% mean sodium intake reduction from current intake levels would be needed to meet the sodium intake recommended levels for adults in scenario A, B and C, respectively.

Data sources: Baseline scenario sodium mean intake: CCHS-Nutrition 2015 and FLIP 2017; Reformulation scenario: CCHS-Nutrition 2015 and FLIP 2017; Population demographics and mortality data 2019: Statistics Canada CANSIM tables.

Note: Sodium intake and SD data for PRIME were converted into Salt (g) using the following formula: Salt (g) = Sodium (mg)* 2.5/1000 mg.

### Potential health impacts of reducing population sodium intakes

#### Scenario A: Canadians’ ‘reformulation’ to meet Health Canada’s 2016 sodium reduction targets (-17%)

Under the ‘reformulation’ scenario A, which resulted in an approximate 17% sodium reduction intake from current levels, overall, 2,176 (95% UI 869–3,687) deaths from CVDs could have been averted or delayed by reducing Canadian adults’ mean sodium intake to 2,300 mg/d–through a systematic reduction in the sodium content of processed foods to meet the 2016 Health Canada’s sodium reduction targets. Approximately, 60% of averted or delayed CVD deaths were observed in men (1,302 [95% UI 508–2,228]) and 40% in women (865 [95% UI 331–1,443]) **(Table 4)**.

**Table 4:** Estimated number of deaths that could be averted or delayed if Canadians were to meet the interim mean sodium intake target of 2,300 mg/day (corresponding to 5.75 g of salt/d) due to a systematic reduction of sodium levels in the Canadian food supply (scenario A)–*presented by cause of death (95% UI)*.

*Cause of death (ICD-10 Code)* ^1^	*Total n (95% UI)* ^2^	*%*	*Men n (95% UI)* ^2^	*%*	*Women n (95% UI)* ^2^	*%*
Cardiovascular diseases	2176 (869, 3687)	100	1302 (508, 2228)	100	865 (331, 1443)	100
Ischaemic heart diseases (CHD) (I20-25)	975 (400, 1635)	44.8	659 (263, 1112)	50.6	312 (121, 512)	36.1
Cerebrovascular diseases (Stroke) (I60-69)	454 (182, 767)	20.9	244 (97, 417)	18.7	207 (81, 343)	23.9
Heart failure (I50)	247 (99, 427)	11.4	129 (50, 227)	9.9	115 (44, 200)	13.3
Aortic aneurysm (I71)	59 (23, 105)	2.7	40 (15, 70)	3.1	19 (7, 34)	2.2
Pulmonary embolism (I26)	12 (4, 26)	0.6	6 (2, 13)	0.5	6 (2, 12)	0.7
Rheumatic heart disease (I05-09)	10 (3, 22)	0.5	4 (1, 9)	0.3	6 (2, 13)	0.7
Hypertensive disease (I10-15)	413 (153, 750)	19.0	213 (77, 389)	16.4	197 (71, 348)	22.8
*Total deaths prevented under age 75*	*795 (308*, *1371)*	*36*.*5*	*585 (224*, *1016)*	*44*.*9*	*205 (78*, *343)*	*23*.*7*
**% of CVD deaths that could have been averted or delayed (reference year: 2019)** ^**3**^	**3.7%**		**4.2%**		**3.1%**	

1. WHO, International Statistical Classification of Diseases and Related Health Problems, Tenth Revision [70].

2. 95% UI are based on 10,000 iterations of Monte Carlo analysis.

3. Deaths in Canada (2019) attributable to the CVDs under study = 58,476 (men 30,663; women 27,813).

Note: total deaths averted or delayed represent less than the sum of its components, given that double counting has been accounted for in PRIME during the modelling process.

Of the total CVD deaths that could have been prevented or delayed, 44.8% (975 [95% UI 400–1,635]) were related to ischaemic heart diseases, followed by stroke 20.9% (454 [95% UI 182–767]), hypertensive disease 19.0% (413 [95% UI 153–750]), heart failure 11.4% (247 [95% UI 99–427]), and aortic aneurysm 2.7% (59 [95% UI 23–105]). Furthermore, 36.5% of potential deaths averted or delayed would be in individuals under 75 years old.

#### Scenario B: WHO sodium recommended levels (-28%)

If the mean population intakes of Canadians would meet the WHO sodium intake recommendation (2000 mg/d), which would mean a 28% sodium reduction intake from current levels, overall, 3,252 (95% UI 1,380–5,321) deaths from CVDs could have been averted or delayed in Canada. Proportion of CVD deaths that could have been averted or delayed are similar than in scenario A **(Table 5)**.

**Table 5:** Estimated number of deaths that could be averted or delayed if Canadians were to meet the WHO mean sodium intake recommendation of 2,000 mg/day (corresponding to 5.00 g of salt/d) (scenario B)–*presented by cause of death (95% UI)*.

*Cause of death (ICD-10 Code)* ^1^	*Total n (95% UI)* ^2^	*%*	*Men n (95% UI)* ^2^	*%*	*Women n (95% UI)* ^2^	*%*
Cardiovascular diseases	3252 (1380, 5321)	100	1899 (776, 3023)	100	1359 (583, 2174)	100
Ischaemic heart diseases (CHD) (I20-25)	1492 (633, 2437)	45.9	997 (406, 1589)	52.5	498 (215, 796)	36.6
Cerebrovascular diseases (Stroke) (I60-69)	689 (292, 1134)	21.2	360 (147, 578)	19.0	329 (140, 528)	24.2
Heart failure (I50)	379 (159, 630)	11.7	192 (80, 315)	10.1	186 (79, 306)	13.7
Aortic aneurysm (I71)	90 (38, 153)	2.8	59 (23, 100)	3.1	31 (13, 52)	2.3
Pulmonary embolism (I26)	19 (6, 39)	0.6	10 (3, 20)	0.5	9 (3, 19)	0.7
Rheumatic heart disease (I05-09)	16 (4, 34)	0.5	6 (2, 13)	0.3	10 (3, 20)	0.7
Hypertensive disease (I10-15)	568 (237, 955)	17.5	271 (110, 438)	14.3	294 (123, 485)	21.6
*Total deaths under age 75*	*1154 (487*, *1885)*	*35*.*5*	*842 (339*, *1351)*	*44*.*3*	*314 (134*, *501)*	*23*.*1*
**% of CVD deaths that could have been averted or delayed (reference year: 2019)** ^**3**^	**5.6%**		**6.2%**		**4.9%**	

1. WHO, International Statistical Classification of Diseases and Related Health Problems, Tenth Revision [70].

2. 95% UI are based on 10,000 iterations of Monte Carlo analysis.

3. Deaths in Canada (2019) attributable to the CVDs under study = 58,476 (men 30,663; women 27,813)

Note: total deaths averted or delayed represent less than the sum of its components, given that double counting has been accounted for in PRIME during the modelling process. WHO’s sodium intake recommendations are directed to individuals and not to the population average, however, for this study we assumed recommendations at the population level.

#### Scenario C: Adequate Intakes recommended levels (-46%)

If the mean population intakes of Canadians would meet the AI for sodium intake (1500 mg/d), which would mean a 46% sodium reduction intake from current levels, overall, 5,296 (95% UI 2,190–8,311) deaths from CVDs could have been averted or delayed in Canada. Proportion of CVD deaths disaggregated by type of CVD and sex that could have been averted or delayed are similar than in scenario A **(Table 6)**.

**Table 6:** Estimated number of deaths that could be averted or delayed if Canadians were to meet the sodium Adequate Intake (AI) recommendation of 1,500 mg/day (corresponding to 3.75 g of salt/d) (scenario C)–*presented by cause of death (95% UI)*.

*Cause of death (ICD-10 Code)* ^1^	*Total n (95% UI)* ^2^	*%*	*Men n (95% UI)* ^2^	*%*	*Women n (95% UI)* ^2^	*%*
Cardiovascular diseases	5296 (2190, 8311)	100	3069 (1296, 4897)	100	2195 (911, 3486)	100
Ischaemic heart diseases (CHD) (I20-25)	2452 (1008, 3845)	46.3	1623 (687, 2587)	52.9	813 (340, 1291)	37.0
Cerebrovascular diseases (Stroke) (I60-69)	1127 (458, 1783)	21.3	582 (247, 933)	19.0	537(223, 858)	24.5
Heart failure (I50)	618 (252, 1005)	11.7	312 (128, 508)	10.2	300 (122, 492)	13.7
Aortic aneurysm (I71)	149 (59, 242)	2.8	96 (39, 160)	3.1	50 (21, 84)	2.3
Pulmonary embolism (I26)	31 (10, 64)	0.6	16 (5, 33)	0.5	15 (5, 31)	0.7
Rheumatic heart disease (I05-09)	27 (7, 55)	0.5	11 (3, 22)	0.4	16 (5, 33)	0.7
Hypertensive disease (I10-15)	893 (367, 1403)	16.9	425 (181, 672)	13.8	461 (191, 738)	21.0
*Total deaths under age 75*	*1878 (773*, *2945)*	*35*.*5*	*1361 (571*, *2180)*	*44*.*3*	*506 (211*, *800)*	*23*.*1*
**% of CVD deaths that could have been averted or delayed (reference year: 2019)** ^**3**^	**9.1%**		**10.0%**		**7.9%**	

1. WHO, International Statistical Classification of Diseases and Related Health Problems, Tenth Revision [70].

2. 95% UI are based on 10,000 iterations of Monte Carlo analysis.

3. Deaths in Canada (2019) attributable to the CVDs under study = 58,476 (men 30,663; women 27,813)

Note: total deaths averted or delayed represent less than the sum of its components, given that double counting has been accounted for in PRIME during the modelling process. Reference AI values for sodium intake are directed to individuals and not to the population average, however, for this study we assumed recommendations at the population level.

In 2019, there were a total of 58,476 deaths due to CVDs in Canada (men 30,663; women 27,813) [69]; therefore, 3.7% fewer CVD deaths would have occurred in that same year (4.2% in men, 3.1% in women) under the ‘reformulation’ scenario–scenario A (Table 7). Additionally, if WHO recommended sodium intake levels were achieved, 5.6% (6.2% in men, 4.9% in women) of CVD deaths in 2019 could have been averted or delayed in scenario B; and 9.1% (10.0% in men, 7.9% in women) in scenario C **(Table 7)**.

**Table 7:** Summary: Deaths that could have been averted or delayed if Canadians were to reduce their mean sodium intake by approximately 17% (scenario A), 28% (scenario B) and 46% (scenario C) to meet recommended sodium intake levels (95% UI) ^1^.

	*Scenario A*		*Scenario B*		*Scenario C*	
	*Reformulation scenario (2300 mg/day)*		*WHO (2000 mg/ day)*		*AI (1500 mg/day)*	
Sex	Number of deaths averted or delayed	% of actual CVDs mortality in 2019 ^**2**^	Number of deaths averted or delayed	% of actual CVDs mortality in 2019 ^**2**^	Number of deaths averted or delayed	% of actual CVDs mortality in 2019 ^**2**^
Men	1302 (508, 2228)	4.2%	1899 (776, 3023)	6.2%	3069 (1296, 4897)	10.0%
Women	865 (331, 1443)	3.1%	1359 (583, 2174)	4.9%	2195 (911, 3486)	7.9%
**Total**	**2176 (869, 3687)**	**3.7%**	**3252 (1380, 5321)**	**5.6%**	**5296 (2190, 8311)**	**9.1%**

1. 95% UI are based on 10,000 iterations of Monte Carlo analysis.

2. Deaths in Canada (2019) attributable to the CVDs under study = 58,476 (men 30,663; women 27,813)

Note: total deaths averted or delayed represent less than the sum of its components, given that double counting has been accounted for in PRIME during the modelling process.

## Discussion

To the best of our knowledge this is the first study to link representative nutritional food composition data from a current national branded food composition database (FLIP 2017) to dietary data from the CCHS-Nutrition 2015, to garner robust estimates of Canadian’s usual sodium intakes and secondly to model the impact of various sodium reduction interventions on diet and health outcomes. Our study found that on average, Canadians had mean daily sodium intakes at levels above the recommended 2300 mg/day, with the vast majority of respondents, across age and sex groups, consuming sodium at levels above the AI, and most above the CDRR. Sodium intakes were highest in males, irrespective of age, which in part is likely driven by higher food and calorie intakes amongst males as compared to females.

Overall, population mean usual sodium intake estimated in this study using linked FLIP 2017 nutrient composition data onto the CCHS-Nutrition, on average, aligned with that previously published by Health Canada [13] and other researchers [72, 78]. Using actual sodium levels of foods sold on the Canadian marketplace in 2017, older DRI age/sex groups had mean usual sodium intakes above those reported by Health Canada using a generic food database [13]. These findings are not surprising given previous work from our research group which revealed a significantly higher sodium composition among some food categories, for example, ‘meat and alternative products’ in FLIP 2017 database as compared to the FID file [16]. Meat and alternative products are a top sodium contributor to the diet, particularly among older adults [79, 80], for which some of these differences in sodium intakes were found [16]. Such data indicate a need for more robust and frequently updated food composition databases to code for foods reported in the CCHS 24- hour recalls, particularly when reformulation efforts are underway in order to better reflect the current Canadian food supply, as not all food categories were reformulated equally nor do all DRI age/sex groups consume the same amounts of different food groups.

Reassuringly, modeled intakes based on a ‘reformulation’ scenario in line with Health Canada’s 2016 sodium reduction targets resulted in mean usual sodium intakes below (-17%) those estimated based on current nutritional values from the actual food supply. In fact, overall, average daily sodium intakes were found to be just under the population target intake at 2299 mg/day, with similar reductions in mean intakes across age and sex groups. Nevertheless, more than half of males still had sodium intakes above the sodium CDRR, indicating that even if Canada were to meet its population based sodium reduction goals, many individuals would still exceed the CDRR.

An unhealthy diet is one of the major preventable risk factors for a range of NCDs, including CVDs, cancer, diabetes and some other metabolic risk factors for these diseases (i.e., high blood pressure, high blood glucose and overweight and obesity) [81]. A healthy food environment can improve diets and, in turn, decrease the burden of diet-related NCDs [82]. One of the most promising strategies to improve diet is the reformulation of food products–to contain lower levels of nutrients of public health concern (sugars, sodium, and saturated and trans fats) and improve the nutritional profile of packaged foods–given that such a strategy can improve diet without requiring consumer behavior change [83]. Processed foods are the primary source of dietary sodium in Canadians’ diet, accounting for 77% of sodium intake [30].

Furthermore, this NCD macro-simulation modelling study, to the best of our knowledge, is the first study in Canada to estimate the health impact of reducing sodium intake due to a systematic ‘reformulation’ in the sodium content of packaged foods in the Canadian food supply to meet the 2016 Health Canada’s sodium reduction targets [29]. If the reformulation targets would have been met–and assuming people’s food choices would remain the same regardless of changes in sodium content in packaged foods, an important number of CVDs could have been prevented or delayed in Canada. To put results from this scenario in context, the estimated number of CVDs that could be prevented or delayed among Canadian adults represent 3.7% (4.2% in men, 3.1% in women) fewer CVD deaths in 2019, with differences in proportional reduction across CVD subtypes (S3 Table 3.1 in S1 File). From all scenarios tested, the ‘reformulation’ scenario could be the most realistic scenario to achieve, given that it would not require consumer’s behavior change and the food industry has already committed voluntarily to reduce sodium levels in food categories with sodium reduction benchmark targets set by Health Canada (2016) [29]. Canada’s food reformulation strategy and targets also aligns with its commitment of reaching the WHO’s goal of a 30% relative reduction in mean population intake of sodium [18], which according to a recent systematic review no country has met yet [28].

This study suggests that most of the CVD deaths averted or delayed would have occurred in people older than 75 years-of-age, consistent with findings from other countries [48, 55, 56], with no significant differences between men and women overall. PRIME estimated that all scenarios tested would have an impact on all CVDs, but mainly on ischaemic heart disease and stroke, and to a lesser extent on pulmonary embolism and rheumatic heart disease. When looking individually at each CVD, we observed that the greatest difference by sex is on ischemic heart disease, where the difference in deaths that could be averted between men and women almost doubles in all scenarios tested. In both men and women, most of the deaths that could have been averted or delayed with reductions in sodium intake were primarily from ischaemic heart disease, followed by stroke and hypertensive disease. This scenario modelling study also demonstrates that greater reductions in CVD deaths could be achieved with further reductions in sodium intakes. Of note, estimated impacts of sodium intake reduction using PRIME are conservative, as they only account for the CVDs mediated by increased blood pressure, and other disease outcomes associated to excessive sodium intake, such as chronic kidney disease and gastric cancer, are not estimated by the model.

Utilizing similar simulation modelling methods to estimate the impact of reductions in dietary sodium on CVD deaths, Belanger et al. estimated that a total of 3,166 (95% UI 2,616–3,604) CVD deaths could have been averted or delayed in 2004 (5.0%) from reducing Canadians’ mean sodium intake from ~3,400 mg/d (CCHS-2004) to ~2,300 mg/d [41], a reduction in sodium intake of approximately 30%. The ‘reformulation’ scenario (or scenario A) in this study, based on current mean sodium intake levels (2,758 mg/day), represents a 17% sodium intake reduction, which would explain the difference (scenario A: 2,176 (95% UI 869–3,687) CVD deaths; 3.7%), but also indicate the benefits of further reductions in sodium intakes.

Research from Australia similarily modelling the impact of voluntary sodium reduction targets revealed a reduction in sodium intakes by an average of 107 mg/day (3.7% from baseline values of 2908 mg/day to 2801 mg/day), preventing approximately 510 deaths/year equivalent to 1% of all CVD, Chronic Kidney Disease (CKD) and stomach cancers [84]. This smaller reduction in sodium intakes in comparison to what was found in our study is likely attributed, in part, to a less comprehensive set of sodium reduction targets (fewer food categories with set targets) than what was outlined in Canada’s Sodium Reduction Strategy [29]. Work from Costa Rica and Brazil revealed that meeting WHO sodium intake recommended levels (2,000 mg/d) would have prevented or postponed 13% of CVD deaths (750 [95% UI 331–1,160]) (mean sodium intake: ~4000 mg/d) [47], and 15% of CVD deaths (46,651 [95% UI 20,066–71,812]) (mean sodium intake: ~ 3,736 mg/d) [48], respectively. Our results suggest that meeting the WHO’s sodium intake goal would have prevented or postponed 5.8% (3252 [1380, 5321]) of CVD deaths in Canada (mean sodium intake: ~ 2758 mg/d). These differences could be attributed to differential contextual factors such as demographic characteristics, CVD burden, and most importantly the large differences in baseline sodium intakes between these countries and current intakes in Canada.

A recent modelling study from England, one of the countries that first introduced a sodium reduction program in 2003, estimated that population sodium intake reductions observed during the 2000–2018 period (from ~ 3,752 mg/d in 2000 to ~ 3,352 mg/d in 2018) prevented 27,630 premature ischaemic heart disease and 39,250 premature stroke cases [85]. The study also concluded that even with sustained 2018 sodium intake levels, an additional 55,850 premature ischaemic heart disease and 72,110 premature stroke cases could be prevented by 2050 [85]. However, health gains could have been even greater if sodium reduction trends observed during the 2003–2010 period had continued to decline [85, 86]. After implementation of the Public Health Responsibility Deal in 2011 –a public-private partnership that provided greater flexibility to the food industry to set and monitor sodium target levels–sodium intake reductions in England slowed significantly, resulting in excess CVD burden and higher healthcare and societal costs [86].

Furtheremore, in South Africa–one of the few countries that passed legislation for mandatory maximun sodium levels for different key food categories [28]–an impact evaluation of their salt reduction program (first phase, 2015–2019) showed a decrease in salt intake of 1.15 g/day (~460 mg/day of sodium) [87]. Interestingly, sodium intake reductions achieved in South Africa are very similar to our results under the ‘reformulation’ scenario (459 mg/day) if Canadian foods were to fully meet Health Canada’s 2016 sodium reduction targets [29], supporting the feasibility of sodium reduction targets. The results from South Africa also demonstrate the greater effectiveness of mandatory targets compared to voluntary programs, such as that in Canada, in improving diets and health outcomes.

In Canada, the just released final regulations requiring mandatory front-of-pack labeling (FOPL) system [88, 89] utilizing ‘high in’ symbols will likely indirectly further motivate the food industry to reformulate packaged foods, including sodium levels. Foods that meet or exceed a predetermined threshold for sodium, sugars or saturated fat will be required by January 1, 2026 to place a ‘high in’ label on the front-of-package [88]. The purpose of a FOPL system is to assess the nutritional quality of a food or beverage product and display it in a simple, easy to interpret visual form for consumers, and to indirectly motivate the food industry to reformulate food products [90–92]. Evidence from Chile shows that a year after implementation of the Chilean FOPL law (2016), there was a significant decrease in the proportion of packaged foods carrying any ‘high in’ warning label (from 51% to 44%), with the most frequent reductions observed in the proportion of packaged foods displaying a sugar or sodium ‘high in’ warning label [93]. Specifically, the proportion of products ‘high in’ sodium went from 74% to 27% in savory spreads, cheeses, ready-to-eat meals, soups, and sausages [93]–food categories that are also top contributors to sodium intakes in Canada [12].

The present study has a number of strengths and limitations that should be considered in the interpretation of our findings. The baseline and counterfactual scenarios were estimated from a nationally representative sample of the Canadian population (CCHS-Nutrition 2015). Biases related to misreporting (due to recall bias) are often attributed to surveys of this nature; however, CCHS-Nutrition 2015 used the Automated Multiple Pass Method to minimize such misreporting bias [42]. Additionally, the NCI method was used to estimate usual intakes which adjusts for within and between-person variation, controlling for dietary misreporting [71]. Also, to estimate sodium content in packaged foods and sodium intake, a representative branded database of foods and beverages sold in Canada was used (FLIP 2017) [43]. With a national sodium reduction strategy in place, using a branded food database based on actual foods on the market place ensures that we used current sodium compositional data. The linking of packaged food and beverage products from the FLIP database onto dietary intake information from the CCHS-Nutrition also allowed us to model the results of meeting Health Canada’s voluntary benchmark sodium reduction targets (2016) [29]. It should be noted however that this, and other counterfactual scenarios modelled in the current study assumed that food choice would remain consistent irrespective of reduced sodium levels in foods. Taste is well established to be a main factor in food selection [94] and it is conceivable that intakes could be altered if substantial reductions in certain market-leading products were made and taste was noticeably changed. Health Canada’s sodium reduction strategy [29] however outlined a phased, or gradual approach, for reducing sodium in foods that has been documented to be less detectable by consumers [95, 96]. Nevertheless, future studies would benefit from examining the impact of sodium reformulation on actual consumer food purchasing behavior and diets.

We used PRIME to estimate the number of CVD deaths that could have been averted or delayed. The model uses relative risks from robust meta-analyses [39] and has been widely used in different countries [46–64], including Canada [41, 65]. It is a user-friendly, transparent and open access model [39]. The PRIME, as other cross-sectional NCD scenario models, does not incorporate the effect of a time lag between the disease outcome and the exposure [39]. Therefore, it is not clear when the estimates predicted by the model could be expected to be achieved. Additionally, PRIME does not investigate morbidity that could be prevented from changes in NCD behavioral risk factors. The meta-analysis used in PRIME only accounted for age-group as an effect modifier. The strengths and limitations of the model have been previously published [39].

The updated Health Canada voluntary sodium reduction targets for processed foods released in 2021 [32], as well as other complementary initiatives in Canada that aim to improve the Canadian food environment, such as FOPL and proposed restrictions on food marketing to children [88, 97], could potentially improve diets in Canada and additionally contribute to reducing overall population sodium intakes. These actions will be particularly important to protect vulnerable populations such as children and adolescents, as the percentage of Canadians consuming excess sodium increases with age during childhood, and peaks during adolescence and early adulthood [13]. Public health experts suggest that, given the slow rate of progress and lack of accountability from the food industry with voluntary targets in different jurisdictions, a mandatory approach could be more effective to ensure food industry compliance in meeting food reformulation targets [24, 27, 98–100]. Despite some progress in reducing sodium in the food supply in Canada [17, 30], independent monitoring, and food industry’s continued efforts to reduce sodium content in the food supply are needed.

## Conclusion

Canadians are consistently consuming high levels of sodium, particularly in certain age and sex groups. We show that reducing dietary sodium intake, a proven cost-effective intervention, could save many lives in Canada by preventing and reducing the burden of CVDs, under different sodium reduction scenarios. Reduced sodium intakes could be achieved through reformulation of the Canadian food supply to meet the targets set by Health Canada. However, it will require higher compliance from the food industry to meet Health Canada’s voluntary benchmark sodium reduction targets. Our results also lend weight to the need for further policy action, such as FOPL, to reduce Canadians’ sodium intake.

## Supporting information

S1 File (DOCX)
