# Supplementary material for: Estimating Canadian sodium intakes and the health impact of meeting national and WHO recommended sodium intake levels: A macrosimulation modelling study
Source: PLoS One. 2023 May 10;18(5):e0284733. doi: 10.1371/journal.pone.0284733 (PMC10171671; doi:10.1371/journal.pone.0284733)
Supplement: S1 File — (DOCX) [file pone.0284733.s001.docx]

**Supporting information**

**Estimating Canadian sodium intakes and the health impact of meeting national and WHO recommended sodium intake levels: a macrosimulation modelling study**

Nadia Flexner^1¶^, Anthea K. Christoforou^1¶^, Jodi T. Bernstein^1^, Alena P. Ng^1^, Yahan Yang^1^, Eduardo A. Fernandes Nilson^2^, Marie-Ève Labonté^3^, *Mary R. L’Abbe^1^

^1^Department of Nutritional Sciences, University of Toronto, Toronto, Ontario, Canada.

^2^Center for Epidemiological Research on Health and Nutrition, University of São Paulo, São Paulo, State of São Paulo Brazil.

^3^Centre Nutrition, Santé et Société (NUTRISS), Institute of Nutrition and Functional Foods (INAF), Laval University, Quebec City, Quebec, Canada

^¶^These authors contributed equally to this work.

*Corresponding author

Email: [mary.labbe@utoronto.ca](mailto:mary.labbe@utoronto.ca)

Contents

[S1 Table 1. Age- and sex-specific estimates of the Canadian population, 2019 2](#_Toc132371211)

[S2 Table 2. Age- and sex-specific estimates of the annual number of CVDs deaths in Canada, 2019 3](#_Toc132371212)

[**S3 Table 3. Proportion of CVD deaths that could have been averted or delayed taking as reference CVD deaths in Canada in 2019 – by CVD subtypes.** 4](#_Toc132371213)

**S1 Table 1. Age- and sex-specific estimates of the Canadian population, 2019 [1]**

**S2 Table 2. Age- and sex-specific estimates of the annual number of CVDs deaths in Canada, 2019 [2]**

| **Males** | **20-24** | **25-29** | **30-34** | **35-39** | **40-44** | **45-49** | **50-54** | **55-59** | **60-64** | **65-69** | **70-74** | **75-79** | **80-84** | **85+** | **Total** |
| --- | --- | --- | --- | --- | --- | --- | --- | --- | --- | --- | --- | --- | --- | --- | --- |
| **I60-I69: Cerebrovascular diseases** | 3 | 7 | 14 | 22 | 30 | 72 | 101 | 190 | 280 | 452 | 641 | 829 | 1,018 | 2,288 | **5,947** |
| **I20-I25: Ischaemic heart diseases** | 2 | 8 | 17 | 65 | 122 | 269 | 573 | 1,049 | 1,631 | 1,999 | 2,324 | 2,394 | 2,691 | 5,626 | **18,770** |
| **I10-I15: Hypertensive disease** | 0 | 0 | 5 | 6 | 14 | 27 | 58 | 97 | 117 | 136 | 156 | 167 | 244 | 620 | **1,647** |
| **I50: Heart failure** | 0 | 2 | 1 | 0 | 9 | 4 | 35 | 47 | 95 | 119 | 232 | 293 | 480 | 1,534 | **2,851** |
| **I71: Aortic aneurysm** | 1 | 1 | 6 | 11 | 12 | 22 | 32 | 41 | 79 | 90 | 141 | 104 | 144 | 260 | **944** |
| **I26: Pulmonary embolism** | 2 | 3 | 2 | 1 | 4 | 10 | 15 | 27 | 22 | 25 | 47 | 43 | 31 | 59 | **291** |
| **I05-09: Rheumatic heart disease** | 0 | 0 | 1 | 2 | 4 | 3 | 5 | 6 | 10 | 9 | 29 | 34 | 35 | 75 | **213** |

| **Females** | **20-24** | **25-29** | **30-34** | **35-39** | **40-44** | **45-49** | **50-54** | **55-59** | **60-64** | **65-69** | **70-74** | **75-79** | **80-84** | **85+** | **Total** |
| --- | --- | --- | --- | --- | --- | --- | --- | --- | --- | --- | --- | --- | --- | --- | --- |
| **I60-I69: Cerebrovascular diseases** | 3 | 7 | 16 | 19 | 26 | 56 | 89 | 138 | 221 | 322 | 516 | 746 | 1,044 | 4,494 | **7,697** |
| **I20-I25: Ischaemic heart diseases** | 1 | 1 | 3 | 14 | 32 | 70 | 142 | 263 | 511 | 717 | 1,067 | 1,216 | 1,845 | 7,230 | **13,112** |
| **I10-I15: Hypertensive disease** | 0 | 0 | 0 | 2 | 7 | 14 | 15 | 27 | 61 | 92 | 124 | 166 | 259 | 1,418 | **2,185** |
| **I50: Heart failure** | 0 | 2 | 1 | 2 | 3 | 4 | 6 | 26 | 43 | 77 | 159 | 243 | 415 | 2,465 | **3,446** |
| **I71: Aortic aneurysm** | 0 | 0 | 1 | 1 | 2 | 3 | 7 | 7 | 18 | 38 | 60 | 84 | 109 | 284 | **614** |
| **I26: Pulmonary embolism** | 3 | 2 | 1 | 6 | 8 | 6 | 11 | 18 | 22 | 36 | 45 | 44 | 40 | 115 | **357** |
| **I05-09: Rheumatic heart disease** | 1 | 1 | 1 | 2 | 3 | 3 | 6 | 9 | 12 | 29 | 31 | 51 | 63 | 190 | **402** |

**S3 Table 3. Proportion of CVD deaths that could have been averted or delayed taking as reference CVD deaths in Canada in 2019 – by CVD subtypes.**

| **S**3 **Table 3.1.** Scenario A: a systematic reduction of the sodium content in packaged foods to meet population mean sodium intake target (2,300 mg/d) | | | | | | |
| --- | --- | --- | --- | --- | --- | --- |
| *Cause of death (ICD-10 Code)* **^1^** | Estimated number of deaths that could be averted or delayed  *Total*  *n (95% UI)* ^2^ | % of CVD deaths that could have been averted or delayed (reference year: 2019) ^3^ | Estimated number of deaths that could be averted or delayed  *Men*  *n (95% UI)* ^2^ | % of CVD deaths that could have been averted or delayed (reference year: 2019) ^3^ | Estimated number of deaths that could be averted or delayed  *Women*  *n (95% UI)* ^2^ | % of CVD deaths that could have been averted or delayed (reference year: 2019) ^3^ |
| **Cardiovascular diseases** | **2176 (869, 3687)** | **3.7** | **1302 (508, 2228)** | **4.2** | **865 (331, 1443)** | **3.1** |
| Ischaemic heart diseases (CHD) (I20-25) | 975 (400, 1635) | 3.1 | 659 (263, 1112) | 3.5 | 312 (121, 512) | 2.4 |
| Cerebrovascular diseases (Stroke) (I60-69) | 454 (182, 767) | 3.3 | 244 (97, 417) | 4.1 | 207 (81, 343) | 2.7 |
| Heart failure (I50) | 247 (99, 427) | 3.9 | 129 (50, 227) | 4.5 | 115 (44, 200) | 3.3 |
| Aortic aneurysm (I71) | 59 (23, 105) | 3.8 | 40 (15, 70) | 4.2 | 19 (7, 34) | 3.1 |
| Pulmonary embolism (I26) | 12 (4, 26) | 1.9 | 6 (2, 13) | 2.1 | 6 (2, 12) | 1.7 |
| Rheumatic heart disease (I05-09) | 10 (3, 22) | 1.6 | 4 (1, 9) | 1.9 | 6 (2, 13) | 1.5 |
| Hypertensive disease (I10-15) | 413 (153, 750) | 10.8 | 213 (77, 389) | 12.9 | 197 (71, 348) | 9.0 |

| **S**3 **Table 3.2.** Scenario B: meeting the WHO recommendation of sodium intake (2,000 mg/d) | | | | | | |
| --- | --- | --- | --- | --- | --- | --- |
| *Cause of death (ICD-10 Code)* **^1^** | Estimated number of deaths that could be averted or delayed  *Total*  *n (95% UI)* ^2^ | % of CVD deaths that could have been averted or delayed (reference year: 2019) ^3^ | Estimated number of deaths that could be averted or delayed  *Men*  *n (95% UI)* ^2^ | % of CVD deaths that could have been averted or delayed (reference year: 2019) ^3^ | Estimated number of deaths that could be averted or delayed  *Women*  *n (95% UI)* ^2^ | % of CVD deaths that could have been averted or delayed (reference year: 2019) ^3^ |
| **Cardiovascular diseases** | **3252 (1380, 5321)** | **5.6** | **1899 (776, 3023)** | **6.2** | **1359 (583, 2174)** | **4.9** |
| Ischaemic heart diseases (CHD) (I20-25) | 1492 (633, 2437) | 4.7 | 997 (406, 1589) | 5.3 | 498 (215, 796) | 3.8 |
| Cerebrovascular diseases (Stroke) (I60-69) | 689 (292, 1134) | 5.0 | 360 (147, 578) | 6.1 | 329 (140, 528) | 4.3 |
| Heart failure (I50) | 379 (159, 630) | 6.0 | 192 (80, 315) | 6.7 | 186 (79, 306) | 5.4 |
| Aortic aneurysm (I71) | 90 (38, 153) | 5.8 | 59 (23, 100) | 6.3 | 31 (13, 52) | 5.0 |
| Pulmonary embolism (I26) | 19 (6, 39) | 2.9 | 10 (3, 20) | 3.4 | 9 (3, 19) | 2.5 |
| Rheumatic heart disease (I05-09) | 16 (4, 34) | 2.6 | 6 (2, 13) | 2.8 | 10 (3, 20) | 2.5 |
| Hypertensive disease (I10-15) | 568 (237, 955) | 14.8 | 271 (110, 438) | 16.5 | 294 (123, 485) | 13.5 |

| **S**3 **Table 3.3.** Scenario C: meeting the Adequate Intake (AI) recommendation of sodium intake for adults (1,500 mg/d) | | | | | | |
| --- | --- | --- | --- | --- | --- | --- |
| *Cause of death (ICD-10 Code)* **^1^** | Estimated number of deaths that could be averted or delayed  *Total*  *n (95% UI)* ^2^ | % of CVD deaths that could have been averted or delayed (reference year: 2019) ^3^ | Estimated number of deaths that could be averted or delayed  *Men*  *n (95% UI)* ^2^ | % of CVD deaths that could have been averted or delayed (reference year: 2019) ^3^ | Estimated number of deaths that could be averted or delayed  *Women*  *n (95% UI)* ^2^ | % of CVD deaths that could have been averted or delayed (reference year: 2019) ^3^ |
| **Cardiovascular diseases** | **5296 (2190, 8311)** | **9.1** | **3069 (1296, 4897)** | **10.0** | **2195 (911, 3486)** | **7.9** |
| Ischaemic heart diseases (CHD) (I20-25) | 2452 (1008, 3845) | 7.7 | 1623 (687, 2587) | 8.6 | 813 (340, 1291) | 6.2 |
| Cerebrovascular diseases (Stroke) (I60-69) | 1127 (458, 1783) | 8.3 | 582 (247, 933) | 9.8 | 537(223, 858) | 7.0 |
| Heart failure (I50) | 618 (252, 1005) | 9.8 | 312 (128, 508) | 10.9 | 300 (122, 492) | 8.7 |
| Aortic aneurysm (I71) | 149 (59, 242) | 9.6 | 96 (39, 160) | 10.2 | 50 (21, 84) | 8.1 |
| Pulmonary embolism (I26) | 31 (10, 64) | 4.8 | 16 (5, 33) | 5.5 | 15 (5, 31) | 4.2 |
| Rheumatic heart disease (I05-09) | 27 (7, 55) | 4.4 | 11 (3, 22) | 5.2 | 16 (5, 33) | 4.0 |
| Hypertensive disease (I10-15) | 893 (367, 1403) | 23.3 | 425 (181, 672) | 25.8 | 461 (191, 738) | 21.1 |

1. WHO, International Statistical Classification of Diseases and Related Health Problems, Tenth Revision [3].
2. 95% UI are based on 10,000 iterations of Monte Carlo analysis.
3. Deaths in Canada (2019) attributable to the CVDs under study = 58,476 (men 30,663; women 27,813)

Note: total deaths averted or delayed represent less than the sum of its components, given that double counting has been accounted for in PRIME during the modelling process. WHO’s sodium intake recommendations and reference AI values for sodium intake are directed to individuals and not to the population average, however, for this study we assumed recommendations at the population level.

**References**

1. Statistics Canada. Table 17-10-0005-01 Population estimates on July 1st, by age and sex 2019 [Available from: <https://www150.statcan.gc.ca/t1/tbl1/en/tv.action?pid=1710000501>.

2. Statistics Canada. Table 13-10-0147-01 Deaths, by cause, Chapter IX: Diseases of the circulatory system (I00 to I99) 2019 [Available from: <https://www150.statcan.gc.ca/t1/tbl1/en/tv.action?pid=1310014701>.

3. World Health Organization. International Classification of Diseases 10 2016 [Available from: <https://icd.who.int/browse10/2016/en#/I20-I25>.
